# Gender differences among board-certified general surgeons in Israel: demographics, professional advancement, and leadership

**DOI:** 10.1186/s13584-026-00787-8

**Published:** 2026-10-27

**Authors:** Shimrit Eliyahu, Yehuda Hershkovitz, Ortal Yzhaky Shapira

**Affiliations:** 1 Department of General Surgery, Edith Wolfson Medical Center, Holon, Israel affiliated with Gray Faculty of Medical & Health Sciences, Tel-Aviv University, Tel-Aviv, Israel; 2 Department of General Surgery, Shamir Medical Center, Zerifin, Israel affiliated with Gray Faculty of Medical & Health Sciences, Tel-Aviv University, Tel- Aviv, Israel

## Abstract

**Background:**

Women remain underrepresented in general surgery leadership and academic roles globally, yet specialty-specific data from Israel are limited. This study examines gender differences among board-certified general surgeons in Israel across demographic, professional, and leadership domains.

**Methods:**

A cross-sectional, confidential questionnaire was distributed electronically to all board-certified general surgeons registered with the Israeli Surgical Association in 2026. A total of 105 surgeons responded (15.2%). Participants were stratified by gender and compared across demographic characteristics, employment patterns, operative scope, fellowship training, academic activity, and management roles.

**Results:**

Of 105 respondents, 78 (74.3%) were men and 27 (25.7%) were women. Women had a longer residency duration (median 7 vs. 6 years, *p* = 0.024). Women were less likely to hold full-time public hospital positions (63.0% vs. 84.6%, *p* = 0.029), participate in on-call (44.4% vs. 80.8%, *p* = 0.0009) or senior on-call duties (7.4% vs. 38.5%, *p* = 0.003), and had a substantially narrower operative scope (median 3 vs. 5 surgery types, *p* = 0.0002). Women were significantly more likely to practice breast surgery (48.1% vs. 16.7%, *p* = 0.002) and less likely to have completed a fellowship (59.3% vs. 84.6%, *p* = 0.013) or trained abroad (25.9% vs. 62.8%, *p* = 0.0015). Academic productivity was also lower among women: fewer taught students (74.1% vs. 91.0%, *p* = 0.044), published articles (29.6% vs. 65.4%, *p* = 0.002), or held academic appointments (25.9% vs. 50.0%, *p* = 0.042). Women were less likely to hold any management role (25.9% vs. 51.3%, *p* = 0.026).

**Conclusions:**

Significant gender disparities exist among board-certified general surgeons in Israel across all domains examined. Women bear a systematically different professional burden, with less call coverage, narrower operative scope, fewer fellowships, lower academic output, and reduced leadership representation. These findings highlight structural barriers that persist beyond training completion. Of particular concern is the leadership gap: in the Israeli hospital system, senior positions are long-tenured and turn over infrequently, meaning that generational change in the trainee demographic alone will not close this gap within any reasonable timeframe. Mandatory, policy-level structural intervention is required alongside broader institutional efforts to promote gender equity in Israeli general surgery.

## Introduction

Gender equity has become a major issue in surgical workforce planning, yet women remain underrepresented in general surgery and especially in leadership and academic advancement [1–3]. Although the proportion of women in medicine has increased steadily in many countries, this growth has not translated evenly into surgical careers, where women continue to face barriers related to operative exposure, schedule control, promotion, and leadership opportunities [2–5].

In Israel, women comprise a substantial share of medical trainees and younger physicians, but their representation decreases in older age groups, full-time clinical work, and senior positions [6, 7]. National workforce data also indicate that female physicians work fewer weekly hours on average than male physicians, and earlier Israeli research has documented gender differences in career pattern, family structure, and productivity [6–8]. However, data specifically focused on board-certified general surgeons in Israel remain limited, particularly regarding fellowship training, operative case distribution, on-call participation, academic output, and representation in management roles.

This gap is significant because surgical advancement depends on these same factors collectively, not training completion alone [1, 3, 4]. International studies show that women surgeons remain underrepresented in surgical society leadership and academic ranks, even as their presence in training programs has grown [2–5]. Qualitative and mixed-methods research further points to institutional culture, mentoring gaps, family responsibilities, and unequal access to sponsorship and promotion as recurring barriers to advancement [4, 5].

Accordingly, the present study examines gender differences among board-certified general surgeons in Israel across three domains: demographics, professional advancement, and representation in leadership or management positions. By describing these patterns in a national cohort, the study aims to provide specialty-specific evidence that may inform surgical workforce planning, mentorship, and equity-oriented policy in Israel.

## Methods

### Study design and participants

This was a cross-sectional, confidential, questionnaire-based study of board-certified general surgeons in Israel, conducted in 2026. The questionnaire was distributed electronically to all 700 board-certified general surgeons registered with the Israeli Surgical Association, via the association’s mailing list and affiliated WhatsApp groups. A total of 105 surgeons completed the questionnaire, yielding a response rate of 15.2%. Participants identified themselves using their medical license number. The study was approved by institutional review board (ASF-0126-25). Completion and submission of the questionnaire were considered the informed consent.

#### Questionnaire development

The questionnaire was developed specifically for this study, as no validated instrument addressing gender differences in surgical career development among Israeli general surgeons was identified in the literature. It was designed to evaluate the personal and professional characteristics of practicing general surgeons and to identify potential gender-related differences in career development, and included demographic, family, and professional sections, detailed below.

#### Demographic and family variables

Demographic information included age, gender, marital status (married/in a relationship, divorced, or single), and number of children. The occupation of the participant’s spouse or partner was also recorded and categorized as full-time employment, part-time employment, full-time employment with high workload, or not employed.

### Employment setting and workload

Because the Israeli healthcare system combines a public hospital system with opportunities for private practice, participants were asked to report their primary employment setting (public hospital, public outpatient clinic, private practice, or unemployed). The primary workplace was defined as the setting in which the surgeon spent more than 50% of their professional activity. Employment within the public healthcare system was further categorized as full-time, part-time, or not employed; this categorization served as the operational measure of workload in the present study. Participants were also asked whether they maintained a private practice and whether they performed surgical procedures in the private sector.

### Additional in-hospital duties

Additional in-hospital duties were assessed, including participation in regular on-call shifts, backup on-call shifts, 24–26-hour overnight shifts, and half-night (8-hour) shifts. In Israel, half-night shifts are typically assigned to surgeons during their first five years following board certification.

### Academic activity and leadership

Academic activity was assessed by academic rank (clinical instructor, lecturer, senior lecturer, or professor), publication of peer-reviewed articles during the previous two years, and involvement in undergraduate medical student teaching during the previous two years. In Israel, undergraduate clinical teaching is considered an integral part of routine clinical practice and is generally performed without additional financial compensation. Leadership positions were categorized as head of service, head of unit, department chair, or surgical ward director.

### Fellowship training

Fellowship training was categorized according to location (Israel, abroad, or research fellowship), subspecialty, and duration.

### Surgical case volume and procedure type

Participants reported the types of surgical procedures they had performed during the previous two years. Minor surgical procedures were defined as abscess drainage, proctologic procedures, and excisional biopsy. Major and complex surgical procedures were defined as trauma surgery, foregut surgery, colectomy, laparoscopic cholecystectomy, hernia repair, and other emergency-oriented procedures.

### Statistical analysis

Descriptive statistics were used to summarize demographic, professional, and academic characteristics of the study sample, stratified by gender. Continuous variables were assessed for normality using the Shapiro-Wilk test; as most continuous variables deviated significantly from a normal distribution, they are presented as medians with interquartile ranges (IQR), and comparisons between men and women were performed using the non-parametric Mann-Whitney U test. Categorical variables are presented as frequencies and percentages. Comparisons between genders were performed using the Chi-square test when all expected cell counts were ≥ 5, and Fisher’s Exact Test when one or more expected cell counts were < 5, including extensions of Fisher’s Exact Test for contingency tables larger than 2 × 2. For variables with several sparsely populated categories, top-level categories were collapsed where clinically appropriate to improve statistical power; in such cases, detailed sub-category counts are presented descriptively alongside the overall test result. For survey items permitting multiple responses (e.g., operative scope, fellowship type), each response option was analyzed as an independent binary variable (present/absent) using Fisher’s Exact Test, since response categories were not mutually exclusive; consequently, no single overall p-value applies across all options within these variables. A two-tailed p-value < 0.05 was considered statistically significant for all analyses.

## Results

### Demographic and career timeline characteristics

The study sample included 105 board-certified general surgeons, 78 men (74.3%) and 27 women (25.7%). The median age of male surgeons was 50 years (IQR 43–59) compared to 46 years (IQR 42–54) for female surgeons; this difference was not statistically significant (*p* = 0.237). The age at residency initiation did not differ significantly between men and women, with both groups demonstrating a median age of 31 years (IQR 30–32 for men, 28–32 for women; *p* = 0.618). By contrast, the duration of residency training was significantly longer among women, with a median of 7.0 years (IQR 6.0–8.0) compared to 6.0 years (IQR 6.0–7.0) among men (*p* = 0.024). The median number of years elapsed since board certification was 11.5 years (IQR 6.0–19.5) for men and 7.0 years (IQR 3.0–16.0) for women, a difference that did not reach statistical significance (*p* = 0.176), though it may reflect the relatively greater representation of women in more recent certification cohorts (Table 1).

**Table 1:** Demographic and family characteristics by gender

Variable	Men (*n* = 78)	Women (*n* = 27)	*p*-value
Age, years, median (IQR)	50 (43–59)	46 (42–54)	0.237
Age at residency initiation, years, median (IQR)	31 (30–32)	31 (28–32)	0.618
Duration of residency, years, median (IQR)	6.0 (6.0–7.0)	7.0 (6.0–8.0)	**0.024**
Years since board certification, median (IQR)	11.5 (6.0–19.5)	7.0 (3.0–16.0)	0.176
***Marital status and family characteristics***			
**Marital status**,** n (%)**			**0.021**
* In a relationship*	72 (92.3)	22 (81.5)	
* Divorced*	5 (6.4)	1 (3.7)	
* Single*	1 (1.3)	4 (14.8)	
* Has at least one child, n (%)*	74 (94.9)	23 (85.2)	0.199
**Number of children among parents onlyᵃ**,** median (IQR)**	**3 (2–3)**	**2 (2–3)**	**0.032**
**Spouse’s career**^**b**^, **n (%)**			0.981
* Full-time employment*	34 (47.2)	10 (45.5)	
* Full-time*,* demanding career*	24 (33.3)	8 (36.4)	
* Part-time employment*	9 (12.5)	2 (9.1)	
* Not employed*	5 (6.9)	2 (9.1)	

IQR, interquartile range. Continuous variables were compared using the Mann-Whitney U test. Categorical variables were compared using the Chi-square test or Fisher’s Exact Test, as appropriate given expected cell counts

ᵃ Restricted to respondents reporting at least 1 child (*n* = 97: 74 men, 23 women) ^b^ Restricted to respondents currently in a relationship (*n* = 94: 72 men, 22 women)

### Marital status and family characteristics

Marital status differed significantly between men and women (*p* = 0.021): 92.3% of men were in a relationship compared to 81.5% of women, and notably, 14.8% of women were single compared to only 1.3% of men. Family size also differed by gender. Among surgeons who were parents, male surgeons had a significantly higher number of children than their female counterparts (median 3 [IQR 2–3] vs. 2 [IQR 2–3]; *p* = 0.032). However, the proportion of surgeons with at least one child did not differ significantly by gender (94.9% of men vs. 85.2% of women; *p* = 0.199), suggesting that the difference lies in family size among parents rather than in the likelihood of having children at all. Among respondents currently in a relationship (*n* = 94), no significant difference was found between men and women in spouse’s career description (*p* = 0.981) (Table 1).

### Work characteristics

No significant gender differences were found in public outpatient clinic employment (42.3% of men vs. 44.4% of women; *p* = 1.000), private clinic practice (43.6% vs. 37.0%; *p* = 0.653), private surgical practice (44.9% vs. 40.7%; *p* = 0.823), or overall main workplace (*p* = 0.318), with the large majority of both groups working primarily in a hospital setting. By contrast, senior position status within the public hospital differed significantly by gender (*p* = 0.029): men were considerably more likely to hold a full-time senior position (84.6% vs. 63.0% of women), while women were disproportionately represented in part-time senior roles (22.2% vs. 6.4% of men). On-call duty patterns showed marked gender differences. Men were significantly more likely to perform on-call duties of any type (80.8% vs. 44.4% of women; *p* = 0.0009), and this gap was even more pronounced for senior on-call duties specifically (38.5% of men vs. 7.4% of women; *p* = 0.0030). Professional subspecialty unit affiliation also differed significantly (*p* = 0.0001): men were substantially more likely to be affiliated with abdominal surgery units (76.6% vs. 40.0% of women), whereas women were markedly overrepresented in breast surgery (45.0% vs. 3.1% of men). Any management role was held by 51.3% of men compared to 25.9% of women (*p* = 0.026), with women underrepresented across all tiers including unit manager (7.4% vs. 25.6%) and department head (3.7% vs. 11.5%) (Table 2).

**Table 2:** Work characteristics by gender

Variable	Men (*n* = 78)	Women (*n* = 27)	*p*-value
**Public hospital senior position, ** ***n*** ** (%)**			0.029
* Full-time*	66 (84.6)	17 (63.0)	
* Part-time*	5 (6.4)	6 (22.2)	
* No*	7 (9.0)	4 (14.8)	
Employed at public outpatient clinic, n (%)	33 (42.3)	12 (44.4)	1.000
Private clinic patients, n (%)	34 (43.6)	10 (37.0)	0.653
Private surgery, n (%)	35 (44.9)	11 (40.7)	0.823
**Main workplace**,** n (%)**			0.318
* Hospital*	73 (93.6)	23 (85.2)	
* Public outpatient clinic*	2 (2.6)	2 (7.4)	
* Private clinic*	2 (2.6)	2 (7.4)	
* Not currently employed*	1 (1.3)	0 (0.0)	
On call duties, n (%)	63 (80.8)	12 (44.4)	**0.0009**
Senior on call duties, n (%)	30 (38.5)	2 (7.4)	**0.0030**
**Professional unit affiliationᵇ**,** n (%)**			**0.0001**
**Abdominal surgery (combined)**	**49 (76.6)**	**8 (40.0)**	
* Bariatric*	14 (21.9)	2 (10.0)	
* Hepatobiliary-pancreatic*	11 (17.2)	1 (5.0)	
* Colorectal*	8 (12.5)	3 (15.0)	
* Abdominal wall*	6 (9.4)	0 (0.0)	
* Esophagogastric*	6 (9.4)	0 (0.0)	
* Otherᶜ*	4 (6.3)	2 (10.0)	
***Breast surgery***	**2 (3.1)**	**9 (45.0)**	
***Trauma / Acute Care***	**13 (20.3)**	**3 (15.0)**	
**Any management role**,** n (%)**	**40 (51.3)**	**7 (25.9)**	**0.026**
* Service manager*	6 (7.7)	3 (11.1)	
* Unit manager*	20 (25.6)	2 (7.4)	
* Department head*	9 (11.5)	1 (3.7)	
* Division head*	5 (6.4)	1 (3.7)	
* No management role*	38 (48.7)	20 (74.1)	

All categorical comparisons were performed using Fisher’s Exact Test

ᵇRestricted to respondents with an assigned professional unit (*n* = 84: 64 men, 20 women); respondents with no unit division were excluded. p-value reflects the overall test across the three top-level categories (Abdominal surgery combined, Breast surgery, Trauma/Acute Care)

ᶜOther includes: endocrine surgery, geriatric surgery, transplant surgery, and oncological surgery

### Operative scope, teaching, and academic activity

Significant gender differences were observed across several core general surgery procedures. Men were significantly more likely to perform esophagogastric surgery (39.7% vs. 7.4% of women; *p* = 0.0015), emergency abdominal surgery (78.2% vs. 44.4%; *p* = 0.0016), colectomy (61.5% vs. 25.9%; *p* = 0.0017), cholecystectomy (82.1% vs. 48.1%; *p* = 0.0018), and hernia repair (80.8% vs. 48.1%; *p* = 0.0022). Conversely, women were significantly more likely to perform breast surgery (48.1% vs. 16.7% of men; *p* = 0.0019). No significant differences were found for trauma surgery or several other procedure types. Overall operative breadth differed significantly: men performed a higher median number of distinct surgery types (5, IQR 4–7) compared to women (3, IQR 1–5) (*p* = 0.0002). With respect to academic and teaching activity, men were significantly more likely to have taught students (91.0% vs. 74.1% of women; *p* = 0.044), published an article in the preceding two years (65.4% vs. 29.6%; *p* = 0.0016), and hold any academic appointment (50.0% vs. 25.9%; *p* = 0.042). Professorship was markedly skewed toward men (19.2% vs. 3.7% of women) (Table 3).

**Table 3:** Operative scope, teaching, academic rank, and publications by gender

Variable	Men (*n* = 78)	Women (*n* = 27)	*p*-value
**Operative Scope - Procedures Performed as Primary Surgeon during the past 2 years**			
Esophagogastric	31 (39.7)	2 (7.4)	**0.0015**
Emergency abdominal surgery	61 (78.2)	12 (44.4)	**0.0016**
Colectomy	48 (61.5)	7 (25.9)	**0.0017**
Cholecystectomy	64 (82.1)	13 (48.1)	**0.0018**
Breast	13 (16.7)	13 (48.1)	**0.0019**
Hernia repair	63 (80.8)	13 (48.1)	**0.0022**
Hepatobiliary-pancreatic	24 (30.8)	3 (11.1)	0.0715
Minor surgery	42 (53.8)	9 (33.3)	0.0771
Proctology	29 (37.2)	5 (18.5)	0.0960
Bariatric	17 (21.8)	2 (7.4)	0.1458
Trauma	1 (1.3)	2 (7.4)	0.1617
Sarcoma/HIPEC	12 (15.4)	1 (3.7)	0.1760
No surgery in past 2 years	1 (1.3)	1 (3.7)	0.4500
Transplant	4 (5.1)	2 (7.4)	0.6458
Endocrine	2 (2.6)	1 (3.7)	1.0000
**Number of surgery types performed**,** median (IQR)**	**5.0 (4.0–7.0)**	**3.0 (1.0–5.0)**	**0.0002**
***Teaching***,*** Academic Rank***,*** and Publications***			
Teaching students, yes	71 (91.0)	20 (74.1)	**0.044**
Published article (past 2 years), yes	51 (65.4)	8 (29.6)	**0.0016**
**Any academic appointment**	**39 (50.0)**	**7 (25.9)**	**0.042**
*Clinical instructor*	11 (14.1)	4 (14.8)	
*Lecturer*	5 (6.4)	1 (3.7)	
*Senior lecturer*	8 (10.3)	1 (3.7)	
*Professor*	15 (19.2)	1 (3.7)	
*None*	39 (50.0)	20 (74.1)	

Operative scope items were analyzed individually using Fisher’s Exact Test, since respondents could select multiple surgery types (not mutually exclusive). Number of surgery types performed (a count variable) was compared using the Mann-Whitney U test. The p-value for academic appointment reflects the collapsed binary comparison (any appointment vs. none); the detailed rank breakdown is shown for descriptive purposes only

### Fellowship training

Fellowship training was significantly more common among men (84.6% vs. 59.3% of women; *p* = 0.013). Men were significantly more likely to have trained abroad (62.8% vs. 25.9% of women; *p* = 0.0015), whereas training within Israel showed a non-significant trend favoring women (37.0% vs. 19.2% of men; *p* = 0.071). Among those who completed a fellowship (*n* = 82: 66 men, 16 women), fellowship duration did not differ significantly (median 15.5 vs. 12.0 months; *p* = 0.782). Fellowship field did not differ significantly overall (*p* = 0.073), although 25.0% of female fellows trained in breast surgery compared to none of the men, while men were more broadly distributed across colorectal, hepatobiliary/transplant, trauma, and bariatric fellowships (Table 4).

**Table 4:** Fellowship training by gender

Variable	Men (*n* = 78)	Women (*n* = 27)	*p*-value
**Fellowship – Any, ** ***n*** ** (%)**	66 (84.6)	16 (59.3)	0.013
**Fellowship typeᵃ**			
Abroad	49 (62.8)	7 (25.9)	**0.0015**
Israel	15 (19.2)	10 (37.0)	0.0713
Research	7 (9.0)	3 (11.1)	0.7147
***Among fellows only (n = 82: 66 men***,*** 16 women)***			
**Fellowship duration**,** months**,** median (IQR)**	**15.5 (12.0–24.0)**	**12.0 (12.0–24.0)**	0.782
**Fellowship field**,** n (%)**			0.073
*Colorectal*	12 (18.2)	3 (18.8)	
*Hepatobiliary/Transplant*	13 (19.7)	2 (12.5)	
*Trauma*	10 (15.2)	3 (18.8)	
*Bariatric*	9 (13.6)	1 (6.2)	
*Esophagogastric*	7 (10.6)	1 (6.2)	
*Breast*	0 (0.0)	4 (25.0)	
*Research*	3 (4.5)	0 (0.0)	
*Abdominal wall*	4 (6.1)	0 (0.0)	
*Minimally invasive surgery*	2 (3.0)	1 (6.2)	
*Pediatric surgery*	2 (3.0)	0 (0.0)	
*Oncological surgery*	2 (3.0)	0 (0.0)	
*Endocrinology*	1 (1.5)	1 (6.2)	
*Thoracic surgery*	1 (1.5)	0 (0.0)	

All categorical comparisons were performed using Fisher’s Exact Test; fellowship duration was compared using the Mann-Whitney U test. Fellowship field p-value reflects the overall Fisher’s Exact Test (2 × 13) among fellows only; sub-category rows are shown for descriptive detail

ᵃMultiple selection was allowed for fellowship type (e.g., clinical abroad and research); percentages are of the full sample (*n* = 78 men, *n* = 27 women) and do not necessarily sum to the “Any” row

## Discussion

According to the most recent report of the Israeli Ministry of Health, women accounted for approximately 20% of the 700 board-certified general surgeons under the age of 67 in Israel as of 2023 — nearly double the 11% recorded in 2010, reflecting a clear generational shift in specialty composition [9]. In our cohort, 25.7% of respondents were women, closely approximating this national figure. This alignment between our sample distribution and the national population proportion supports the representativeness of our data and strengthens the validity of the gender comparisons reported throughout this study.

Our study demonstrates persistent gender disparities among board-certified general surgeons in Israel across demographic, clinical, academic, and leadership domains. Women were less likely to be married or partnered, had fewer children, and had slightly longer residency duration than men. These patterns suggest structural rather than individual explanations for observed disparities — career progression in surgery appears to remain differentially constrained for women during family-building years in ways that compound over time. These findings are consistent with prior peer-reviewed Israeli and international studies showing that women in surgery often face differential training pathways and stronger family-related constraints during career development [10, 11].

The observed differences in work patterns are especially important because workload structure is closely tied to professional advancement in surgery. In our cohort, women were less likely to work full time in public hospitals (63.0% vs. 84.6%, *p* = 0.029) and were substantially less likely to participate in regular on-call shifts (44.4% vs. 80.8%, *p* = 0.0009) or senior on-call shifts (7.4% vs. 38.5%, *p* = 0.003), indicating that men carried a disproportionate share of acute-care responsibilities. Prior studies have shown that women surgeons are more likely to work fewer hours and adjust career choices in response to family demands, which may limit exposure to operative volume, emergency cases, and institutional visibility. Since call burden and sustained hospital presence are often prerequisites for operative exposure, these differences may have downstream effects on fellowship competitiveness and promotion [12, 13]. Addressing this disparity will require more than scheduling reform. Hospital administrations should consider providing institutionally funded childcare and family support services — including on-site or subsidised childcare facilities and backup care provision — for clinical staff at all career stages, from residency through seniority. Evidence indicates that access to such support improves work-life balance, reduces attrition, and enables healthcare professionals to maintain greater focus and engagement with their clinical roles [14]. For female surgeons, whose family management responsibilities disproportionately constrain professional availability, institutional investment in this infrastructure represents a meaningful and tangible commitment to workforce equity.

Gender differences in operative exposure were also pronounced. This distribution is not merely descriptive — it directly shapes operative autonomy, subspecialty identity, referral patterns, and long-term career trajectory in ways that systematically disadvantage women. Men more frequently performed esophagogastric surgeries, emergency abdominal operations, colectomy, cholecystectomy, and hernia procedures, while women were significantly more represented in breast surgery (48.1% vs. 16.7%) and reported a substantially lower operative breadth overall (median 3 surgery types vs. 5, *p* = 0.0002). Notably, trauma surgery rates did not differ significantly between men and women. This pattern is broadly consistent with prior findings on gender disparities in general surgery fellowship subspecialization, in which female enrollment has lagged in several procedure-intensive fellowships even as overall female representation in general surgery residency has increased. Given that operative volume is a primary currency of surgical advancement — determining fellowship competitiveness, referral networks, and perceived surgical identity — these differences in case distribution represent a structural barrier, not a matter of individual preference [15].

The fellowship findings in this study warrant closer examination. Women were significantly less likely to have completed any fellowship (59.3% vs. 84.6%, *p* = 0.013), and among those who did, far fewer trained abroad (25.9% vs. 62.8%, *p* = 0.0015). Among fellows, women were the only group to complete breast surgery fellowships (25.0% vs. 0% of male fellows), while men dominated procedure-intensive subspecialties such as hepatobiliary/transplant, bariatric, and esophagogastric surgery. Fellowship training has been linked to later career advancement, including subspecialty recognition, academic visibility, and leadership opportunities. These patterns may reflect lifestyle considerations that weigh differently for women and men, as well as practical barriers to mobility, family constraints, or unequal access to mentoring and sponsorship in the Israeli context [16–18]. A structural measure with direct applicability is the expansion of part-time fellowship tracks across surgical subspecialties. Completing a fellowship on a reduced-hours basis over an extended period substantially lowers the burden for surgeons with family responsibilities without compromising training depth. This model is not theoretical: the Israeli Medical Association’s Scientific Council already formally recognises both a full-time (12-month) and a part-time (24-month) pathway for the breast surgery fellowship, and a comparable arrangement exists for abdominal wall reconstruction [19]. Extending this framework to additional subspecialties would constitute a concrete policy intervention capable of broadening fellowship access for female surgeons and, over time, diversifying the subspecialty landscape.

Academic productivity and educational engagement were also lower among women in our sample. Women were significantly less likely to teach students (74.1% vs. 91.0%, *p* = 0.044), to have published a peer-reviewed article in the preceding two years (29.6% vs. 65.4%, *p* = 0.0016), and to hold an academic appointment (25.9% vs. 50.0%, *p* = 0.042), with women reaching professorial rank at only 3.7% compared to 19.2% of men. The academic rank finding is particularly notable and may reflect unequal access to academic tracks or the compounding effect of reduced protected time for research among women managing clinical and family responsibilities. This is consistent with broader surgical literature showing persistent gender disparities in academic rank and leadership, even among surgeons with advanced degrees. In a large academic surgery analysis, women were less likely than men to be full professors and held fewer chair and division chief roles, indicating that academic credentials do not automatically translate into equal advancement. Our findings extend this concern to Israeli general surgery and suggest that similar structural barriers may operate locally [20].

The leadership gap in our study is particularly significant from a health-system perspective. Only 25.9% of women held any management role, compared with 51.3% of men (*p* = 0.026), with women markedly underrepresented at higher leadership tiers such as unit manager (7.4% vs. 25.6%) and department head (3.7% vs. 11.5%). These proportions closely mirror the national landscape. Across all general surgery departments in Israeli hospitals, only 3 of 37 department heads (8.1%) are women (one head of general surgery department, and other 2 female surgeons are defined as head of department of breast surgery by their institution), 2 of 28 heads of service (7.1%) are women, and 23 of 111 unit heads (20.7%) are women. The alignment between our cohort data and these national figures suggests that our findings are not a sampling artefact but an accurate reflection of the gender composition of surgical leadership across the Israeli healthcare system [9]. Prior peer-reviewed studies have demonstrated that women remain underrepresented in surgical leadership roles across institutions and societies, despite gradual improvement over time [21, 22]. This underrepresentation is not a peripheral concern. Leadership shapes hiring decisions, fellowship allocation, operative scheduling, mentorship access, and the professional culture that determines whether women surgeons can advance. The absence of women from leadership therefore actively reproduces the disparities observed in operative workload and academic output — creating a self-reinforcing cycle that will not resolve without deliberate institutional intervention. A critical and often overlooked dimension of this problem is the tenure structure of surgical leadership in Israel. Department heads and unit managers are typically appointed for extended terms and routinely remain in post until retirement, resulting in slow turnover and infrequent vacancies. In this environment, a strategy that relies on organic generational change — anticipating that growing female representation in the surgical trainee pool will eventually produce proportionate leadership — is not viable within any meaningful policy timeframe. The pipeline is changing; the positions at its end are not. Closing this gap will require mandatory structural measures: defined gender representation benchmarks for leadership appointments, transparent and accountable selection processes, renewable multi-year terms rather than indefinite tenure, and active succession planning that does not systematically reproduce the current demographic composition of surgical leadership. A concrete and immediately actionable mechanism exists within the Israeli system to begin implementing this change. The Committee for Appointments to Senior Medical Leadership Positions in Hospitals — a statutory body appointed by the Ministry of Health and responsible for recommending candidates for department head and senior unit manager roles in public hospitals — should itself be required to reflect the gender composition of the relevant specialty workforce. In general surgery, where women currently comprise approximately 20% of board-certified surgeons, this would mean ensuring that at least one in five members of the appointing committee is a woman. A gender-representative selection committee is more likely to apply equitable criteria, broaden the scope of candidacy, and actively counteract the informal homophily that tends to perpetuate existing leadership demographics. This reform does not require new legislation — it can be implemented through a ministerial directive and represents a proportionate, evidence-informed, and structurally meaningful first step toward gender equity in Israeli surgical leadership.

An additional structural dimension deserves attention. In our sample, women had a significantly shorter median time since board certification than men (7.0 vs. 11.5 years), reflecting the broader trend of increasing female entry into surgical training in recent years. While this generational shift is encouraging, it also means that women are entering independent surgical practice at a time when the positions that define career advancement — management roles, senior on-call responsibilities, academic appointments, and subspecialty leadership — are predominantly held by a more senior, predominantly male cohort. In the Israeli hospital system, such positions tend to be long-tenured and do not turn over frequently, meaning that the structural landscape women encounter upon completing board certification may remain largely unchanged for years. For newly certified female surgeons, this creates a compounded challenge: not only must they navigate the barriers to advancement documented in this study, but they do so in an environment where the available positions are limited and the incumbents are unlikely to vacate them in the near term. This dynamic suggests that passive generational change will be insufficient to close the gaps observed here, and that active institutional efforts to create opportunities for recently certified surgeons — regardless of gender — will be necessary to enable meaningful advancement.

From a policy standpoint, our findings support the need for structural rather than purely individual solutions. Transparent criteria for leadership promotion, equitable access to operative lists, protected time for research and teaching, and family-sensitive scheduling may help reduce disparities in career progression. In surgery, where advancement depends heavily on cumulative operative exposure and institutional visibility, targeted interventions may be necessary to prevent gender gaps from widening over time. Prior literature also suggests that mentorship, sponsorship, and visible female role models are associated with improved representation of women in surgical training and leadership. Beyond equity, these disparities may carry implications for patient care. Large population-based studies using Medicare and Ontario health system data have reported lower postoperative mortality among patients treated by female surgeons compared with male surgeons, with patient-surgeon gender concordance associated with modestly lower mortality for female patients in some analyses, although the effect sizes were small, and the underlying mechanisms remain unclear [23–25]. Regardless of the precise relationship to outcomes, a surgical workforce in which women are structurally excluded from high-volume operative practice and leadership represents an underuse of trained surgical talent and limits the diversity of perspectives available within surgical care.

This study adds specialty-specific evidence that gender inequity persists in Israeli general surgery well beyond training and into independent practice. Women surgeons in Israel bear a systematically different professional burden — less call, lower operative volume, reduced academic participation, and fewer leadership opportunities — despite holding the same board certification as their male colleagues. These findings are not incidental. They reflect the cumulative effect of scheduling structures, fellowship access, mentorship gaps, and leadership pipelines that were not designed with women’s career trajectories in mind. The surgical community in Israel, its hospitals, and its policymakers have an obligation to move beyond acknowledgment toward action: transparent promotion criteria, equitable operative scheduling, protected research time, and meaningful representation of women in institutional leadership. The women who complete surgical training and earn board certification in Israel have already demonstrated exceptional commitment and competence. The question is whether the system will offer them an equal opportunity to lead.

## Limitations

Several limitations of this study should be considered when interpreting the findings. First, only 105 of 692 registered board-certified general surgeons responded to the questionnaire, representing a response rate of 15.2%. While this rate is not unusual for voluntary physician surveys distributed through professional mailing lists, it raises the possibility of response bias — those who chose to participate may differ systematically from non-respondents in ways we cannot assess. In particular, surgeons with stronger opinions about gender equity, or those with more time available to complete a survey, may have been more likely to respond. Second, the questionnaire was developed specifically for this study and was not based on a previously validated instrument. Although it was designed to capture the key domains of surgical career development, it has not undergone formal psychometric validation, and the wording of individual items may have introduced variability in how respondents interpreted certain questions. Third, all data were self-reported, which introduces the potential for recall bias and social desirability effects, particularly for items relating to operative volume, academic output, and leadership roles. Finally, the relatively small number of female respondents (*n* = 27) limited statistical power for subgroup analyses and should be considered when assessing the precision of effect estimates. In addition, most survey respondents were hospital-based physicians, whereas only a small proportion practiced primarily in the community. This represents a limitation in terms of the representativeness of the sample. However, physicians whose primary workplace is in the community are generally less likely to perform surgery as a routine part of their practice. Therefore, the composition of our sample may more accurately reflect the population of actively practicing surgeons and, as such, may strengthen the relevance of our findings to the research question.

## Conclusion

This study demonstrates that meaningful gender disparities persist among board-certified general surgeons in Israel across multiple professional domains. Women in this cohort were less likely to work full time in public hospitals, participated substantially less in on-call and senior on-call duties, performed a narrower range of surgical procedures, completed fewer fellowships, trained abroad less frequently, published less, held fewer academic appointments, and were underrepresented in management roles at every level. These differences exist among surgeons who have met the same certification standard — they cannot, therefore, be attributed to differences in qualification or commitment. The consistency of findings across so many domains points toward structural explanations: scheduling systems, fellowship pipelines, academic promotion pathways, and leadership selection processes that were not designed with women’s career trajectories in mind, and that continue to produce unequal outcomes even as the composition of the surgical workforce changes. A more systematic and thorough study should be undertaken in this field and in other subspecialities, based on available data, either by the ministry of health or the professional society.

Change is underway: national data show that the proportion of female board-certified general surgeons in Israel has nearly doubled over the past decade, from 11% in 2010 to 20% in 2023. Yet this generational progress has not reached the top — nationally, women hold only 8.1% of general surgery department head positions (3/37), 7.1% of head of service positions (2/28), and 20.7% of unit head positions (23/111) — figures that closely match the leadership gaps documented in our cohort — and leadership positions in Israeli public hospitals are long-tenured and rarely vacated before retirement. Passive demographic change will not close this gap within any reasonable timeframe. Targeted structural intervention is required, at both the institutional and governmental level. Specifically, we recommend the following: first, the Ministry of Health should issue a directive requiring that the Committee for appointments to senior medical leadership positions in hospitals reflects the gender distribution of the relevant specialty. Second, senior leadership appointments should transition to renewable multi-year terms rather than indefinite tenure, creating regular opportunities for advancement. Third, part-time fellowship tracks — already recognised by the Israeli Medical Association for breast surgery and abdominal wall reconstruction — should be extended to additional subspecialties, reducing a structural barrier to fellowship completion for surgeons with family responsibilities. Fourth, hospital administrations should fund on-site or subsidised childcare services for clinical staff, enabling female surgeons to maintain professional availability without bearing a disproportionate domestic burden. These measures are concrete, implementable within the existing regulatory framework, and proportionate to the scale of disparity this study documents. The evidence is clear; what is required now is the institutional will to act.

## Data Availability

No datasets were generated or analysed during the current study.

## Declarations

### Generative AI and AI-assisted technologies in the manuscript preparation process

During the preparation of this work the authors used Claude (Anthropic, San Francisco, CA) service to assist with language editing, text refinement, and structural revision of the manuscript. After using this tool, the authors reviewed and edited the content as needed and take full responsibility for the content of the published article.
